# Effect of respiratory training on respiratory failure secondary to unilateral phrenic nerve injury: A case report

**DOI:** 10.1097/MD.0000000000032566

**Published:** 2023-02-17

**Authors:** Dawei Li, Zhendong Li, Zhiyou Zhang, Yueyang Liu, Congcong Wang, Aixia Cheng

**Affiliations:** a Neurorehabilitation Department, Shengli Oilfield Central Hospital, Dongying, Shandong, China.

**Keywords:** diaphragm, phrenic nerve injury, rehabilitation training, respiratory failure, ventilation therapy

## Abstract

Diaphragm is one of the most important respiratory muscles dominated by the phrenic nerve. Phrenic nerve injury would induce a series of clinical symptoms, including respiratory failure. Respiratory training could assist in regular treatment in improving the respiratory function and daily ability of respiratory failure patients.

**Case presentation:**

A 71-years-old female was enrolled for the disorders of consciousness of 4.5 hours observed by her family and was diagnosed with respiratory failure secondary to unilateral phrenic nerve injury. The patient received basic therapy combined with rehabilitation training, including the training of aspirate muscle, limb resistance, thoracic loosening, aerobic training, electrical stimulation on respiratory nerves, and airway clearance. The combining therapeutic strategy significantly improved the daily ability and respiratory of the patient. The ultrasound showed that after therapy, the diaphragmatic muscles were thickened and the range of diaphragmatic movement was also enhanced. The pulmonary function was also improved after therapy.

**Conclusion:**

The combination of rehabilitation is suitable for the treatment of respiratory failure patients with clear causes of phrenic nerve injury. For patients with unexplained causes, rehabilitation could also be performed before the diagnosis. Patients with irreversible injury need long-term and family rehabilitation prescriptions.

## 1. Introduction

Respiratory failure is a kind of severe lung ventilation impairment induced by various factors, which could further lead to anoxia, retention of CO_2_, and a series of disorders.^[1,2]^ In the clinic, respiratory failure always refers to external respiratory failure, including the ventilation activity of respiratory organs, which are affected by the driving of the respiratory center, thoracic integrity, intact respiratory muscles, intact pleural space, airway patency, and well lung compliance.^[3,4]^

The respiratory muscles are the driving force of breathing, where the diaphragm innervated by the phrenic nerve is the most critical muscle accounting for 75% to 80% of the inhalation processes.^[5,6]^ In the present study, the diagnosis, treatment, and recovery of a case of secondary respiratory failure induced by unilateral phrenic nerve damage were reported.

## 2. Case presentation

This study obtained approval from the Ethic committee of Shengli Oilfield Central Hospital and informed consent had also been obtained from the family of the patient. A 71-years-old female was enrolled for the disorders of consciousness of 4.5 hours observed by her family. The patient had shown the symptoms of cough, sputum production, and wheezing for 5 years, which were recurrent and aggravated in the past month. The patients possessed a history of hypertension, cervical spondylosis, and insomnia. The patients had received the “small needle knife” therapy in the neck for 20 years. The patient was without a history of exposure to chemicals, radioactive, or toxic substances, no history of smoking, and no history of chronic obstructive pulmonary disease. The patient took 2 tablets of diazepam at the night before the onset to help sleep.

The body temperature of the patient was 37.1°C with a blood pressure of 178/101 mm Hg (DBP/SBP) at her admission and the heart rate was 108/minutes and the respiratory rate was 25/minutes. The patient was shallow coma and had occasional eye-opening when feeling tingling pain. The Glasgow coma scale score was 8. Breathing in both lungs was low and the left lower lung was obvious with the scattered wet crisis. No obvious abnormalities in the cranial nerve examination. Stinging of the extremities can be localized. Muscle tone and tendon reflexes are normal, and bilateral pathological signs are not elicited. But the patients did not cooperate in other body examinations. The arterial blood gas analysis (pH = 7.09, O_2_ = 69 mm Hg, CO_2_ = 132 mm Hg, and alkali residual = 4.0 mm) and chest CT showed the bilateral pleural effusion and consolidation of adjacent lung tissues and the left diaphragm swelled, bilateral chest was edematous.

According to the clinical symptoms and results of the auxiliary tests, the patient was finally diagnosed with acute pulmonary encephalopathy, type II respiratory failure, respiratory acidosis, diaphragm elevation, and pulmonary infection on 27^th^ December 2021 and admitted to the neurocritical care unit of Shengli Oilfield Central Hospital.

The patient received basic therapy combined with rehabilitation and was discharged on 16^th^ January 2022. Specifically, according to the history of drug allergies, the patient received antibiotic anti-infection therapy and endotracheal intubation therapy to assist with ventilation, improve respiratory failure, and correct respiratory acidosis. The diuretics were used to control the water-liquid balance, meanwhile, enteral nutrition and venous thromboembolism prevention program were also carried out. The rehabilitation was performed after the consciousness of the patient cleared and throughout the inpatient period of the neonatal intensive care unit and rehabilitation medicine department.

The rehabilitation included the training of aspirate muscle, limb resistance, thoracic loosening, aerobic training, electrical stimulation on respiratory nerves, and airway clearance. The aspirate muscle training was carried out with the help of an inspiratory muscle trainer (Powerbreath^®^, POWER breath International Ltd., UK) with a resistance range of 0 to 90 and a frequency of 15 minutes each time, twice a day, 5 days a week. The limb resistance training included abdominal and lip retraction breathing and the resistance target was set to a lad equivalent to 20% to 40% of the maximum repeat action. The frequency was 8 to 12 times a group, 2 groups a time, twice a day. The frequency of thoracic loosening was 2 to 3 cycles a group, 2 to 3 groups each time, twice a day. The aerobic training included walking and pedaling training. During exercises, the oxygen saturation, heart rate, Borg fatigue, dyspnea scores, and exercise distance were recorded, and the exercise intensity should be 50% to 70% of the maximum heart rate. The electrical stimulation was performed on the superficial bilateral phrenic nerve and the surface of the rectus abdominal muscle with the intensity of 20 to 25 mA for 20 minutes each time, twice a day. The airway clearance was completed independently by the patient’s family after the corresponding mission.

**Figure 1.**
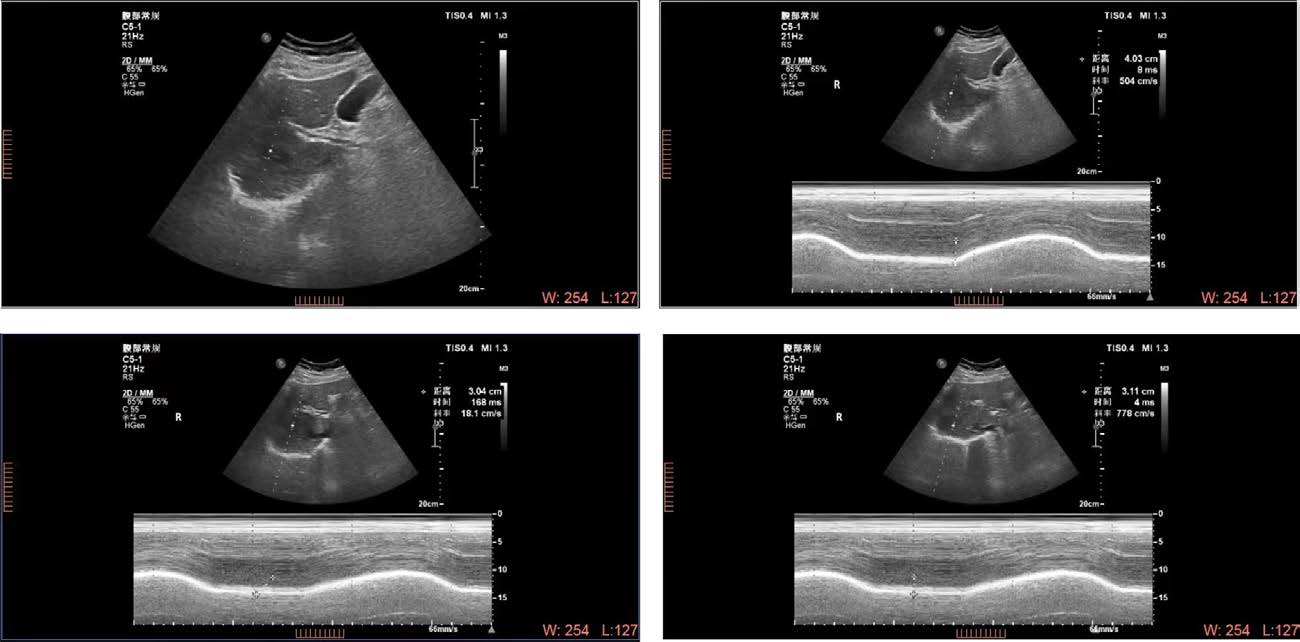
Regular abdominal doppler ultrasound of the patient on admission.

After 21-day hospitalization, the Advanced Decline Line score of the patient reached 100 and the patient was able to complete daily life and some social activities independently when she was discharged. The patient was diagnosed with sleep apnea syndrome caused by obesity and was suggested to wear a noninvasive ventilator to aid breathing during night sleep followed up for 1.5 months. The arterial blood gas analysis results showed that the CO_2_ pressure, standard base excess standard base excess, and HCO_3_^-^ significantly decreased after therapy combing with rehabilitation, while after 1.5 months of follow-up, these indexes were found to be stably reduced (Table 1). The movement amplitude of the bilateral diaphragm was increased when discharged and was persistently elevated during the 1.5-month follow-up. Moreover, the thickening rate of the bilateral diaphragm also increased after therapy (right diaphragm 50% vs 40%, lift diaphragm 11% vs 5%) and the 1.5-month follow-up (right diaphragm 55%, left diaphragm 19%, Fig. 1). For the lung function, both forced vital capacity (FVC, 53.5% vs 18.4%) and forced expiratory volume in the first second (FEV1, 47.7% vs 15.6%) were significantly improved after therapy and the FEV1/FVC ratio was also increased. The 1.5-month follow-up showed that the FVC, FEV1, and the ratio of FEV1/FVC were slightly decreased (Table 2).

**Table 1:** The arterial blood gas analysis of the patient during the study.

	Before therapy	Discharged	1.5 Months of follow up
pH	7.09	7.38	7.37
PCO_2_ (mm Hg)	132	53.5	21.3
PO_2_ (mm Hg)	69	64.4	65.7
HCO_3_^-^ (mmol/L)	40.0	32.1	29.6
ABE (mmol/L)	4.0	5.6	3.1

ABE = standard base excess.

**Table 2:** The indexes evaluating the lung function of the patient during the study.

	Before therapy	Discharged	1.5-Month follow up
FVC (%)	18.4	53.5	52.6
FEV1 (%)	15.6	47.7	46.69
FEV1/FVC (%)	84.3	98.1	96.9

FEV1 = forced expiratory volume in the first second, FVC = forced vital capacity.

## 3. Discussion

Phrenic nerve injury is a newly defined neurological term referring to the neurological dysfunction induced by the damaged phrenic nerve.^[7]^ The major symptoms behaved as the paralyzed ipsilateral hemidiaphragm and weaken or even disappeared abdominal breathing. The phrenic muscle, as the most important respiratory muscle, plays a critical role in the breathing exercise processes, especially in the inhalation process.^[8]^ Pump failure caused by insufficient respiratory drive or movement restriction would result in decreased ventilation, which always presents as hypoxia and CO_2_ retention (respiratory failure type II). The factors that influenced the activity of the phrenic muscles are risk factors for respiratory failure.

The diaphragmic muscles are dominated by the ipsilateral phrenic nerve, which originates from the cervical plexus.^[9]^ The bilateral phrenic nerves are emitted by the central branch of C3 to C5 nerves and were mainly composed of the ventral branch emitted by C4.^[10]^ Usually, the left nerve is longer than the right. The phrenic nerve passes through the neck and the chest cavity, and the phrenic nerve at the lateral border of the anterior oblique muscle forms the main branch, which converges to form the main trunk of the phrenic nerve. Additionally, it could also descend to the thoracic cage to form the backbone of the phrenic nerve. During the travel, it is adjacent to the internal jugular vein, the total jugular/ascending artery, the subclavian artery/vein, the internal thoracic artery, the superior vena cava, the right upper pulmonary vein, the pericardial diaphragm artery, other important organs.^[11]^ In traditional operations, such as cardiac surgery, right upper pulmonary vein intervention, and transdiaphragmatic approach surgery, the phrenic nerve is susceptible to injury. Aphrodisiac injury of 1 side phrenic nerve may cause ipsilateral shoulder radiation pain or mild dyspnea. Some patients may not show obvious symptoms due to the addition of the phrenic nerve and therefore lack of clinical attention.

In the present study, the patient was diagnosed with diaphragmatic palsy secondary to unilateral phrenic nerve near-complete damage, which was considered to be related to neck puncture therapy. The patient suffered from a left phrenic nerve injury in middle age, but the daily respiratory can be maintained through compensatory ventilation of the right phrenic and extracostal muscles. With the increasing age, obesity, and the decreasing compensatory capacity of phrenic muscles, the patient gradually showed the clinical symptoms of respiratory failure. When the occurrence of pulmonary infection and the use of sedatives, the patient ventilation function was totally decompensated, leading to CO_2_ retention secondary to type II respiratory failure and pulmonary encephalopathy.

The therapeutic strategy of the present case was firstly correct respiratory and internal environmental disorders using mechanical ventilation to maintain the synthesis of arterial blood oxygen. It should be noticed that due to obesity, the time of mechanical ventilation should be strictly controlled to prevent ventilator-related respiratory muscle fatigue and to reduce the risk of respiratory muscle atrophy and the difficulty of withdrawal. It was reported that diaphragm dysfunction may occur more often than limb muscle weakness in ICU patients. The prevalence of diaphragm dysfunction in ICU-AW patients during the withdrawal process is as high as 80%.^[12]^ Recently, with the continuous improvement of the theoretical and practical capabilities of respiratory rehabilitation in China, respiratory rehabilitation has become one of the most important interventions for chronic respiratory diseases, which can alleviate clinical symptoms and improve functional outcomes of COPD patients, asthma, bronchiectasis, ILD, PH, lung cancer, and pneumonia to varying degrees.^[13,14]^ However, respiratory rehabilitation was rarely applied in patients similar to the respiratory dysfunction caused by surgery, trauma, and other factors in the present case.

Because phrenic nerve injury is irreversible, the ventilation function was improved mainly through inspiratory muscle training, body resistance training, airway clearance ability training, thoracic mobility, and systemic aerobic metabolism ability training, which could improve the strength and endurance of the contralateral diaphragm and intercostals muscles, guarantee the airway patency, and therefore reduce the occurrence of restrictive ventilation. Meanwhile, part of the expiratory muscles (abdominal muscles and intercostal muscle) was involved in the inspiratory process to exert a compensatory effect. However, it is necessary to prevent excessive participation in expiratory muscle fatigue, decompensation, and other conditions. The changes in diaphragm anatomical position and the movement ability, including the range of motion and the thickening rate, were monitored with ultrasound, while the blood gas analysis was helpful for the diagnosis and treatment of hypoxemia and hypercapnia. The ventilation morphology and function were evaluated with pulmonary function, where the activity of the diaphragm was closely correlated with pulmonary function.^[15,16]^

Taken together, in the treatment of patients with unilateral phrenic nerve and diaphragm injury of clear causes, it is necessary to choose reasonable assessment methods to clarify the status of ventilation function and perform early intervention of ventilation dysfunction via precise breathing training combined with limb training or other comprehensive pulmonary rehabilitation. Patients with unexplained phrenic nerve injury were also suitable for rehabilitation training before diagnosis. For patients who have been diagnosed with irreversible impairments, long-term training plans and family rehabilitation prescriptions should be formulated to reduce a series of secondary impairments, such as respiratory failure secondary to decompensation of chronic ventilation dysfunction.

## Author contributions

**Conceptualization:** Dawei Li, Aixia Cheng.

**Data curation:** Zhendong Li, Yueyang Liu.

**Investigation:** Zhiyou Zhang, Congcong Wang.

**Methodology:** Zhiyou Zhang, Congcong Wang.

**Software:** Yueyang Liu.

**Writing – original draft:** Zhendong Li, Aixia Cheng.

**Writing – review & editing:** Dawei Li, Zhendong Li, Aixia Cheng.
